# Process Evaluation of Lumbar Interbody Fusion Surgeries in Five Dutch Hospitals: A Qualitative Analysis

**DOI:** 10.3390/medicina58010099

**Published:** 2022-01-09

**Authors:** Ruud Droeghaag, Inge J. M. H. Caelers, Aggie T. G. Paulus, Wouter L. W. van Hemert, Henk van Santbrink, on behalf of The LIFT-Study Group

**Affiliations:** 1 CAPHRI School for Public Health and Primary Care, Maastricht University, 6229 ER Maastricht, The Netherlands; inge.caelers@mumc.nl (I.J.M.H.C.); a.paulus@maastrichtuniversity.nl (A.T.G.P.); h.van.santbrink@mumc.nl (H.v.S.); 2 Department of Neurosurgery, Zuyderland Medical Centre, Sittard-Geleen, 6130 MB Heerlen, The Netherlands; 3 Department of Orthopaedic Surgery, Zuyderland Medical Centre, Sittard-Geleen, 6130 MB Heerlen, The Netherlands; w.vanhemert@zuyderland.nl; 4 Department of Neurosurgery, Maastricht University Medical Centre, 6229 HX Maastricht, The Netherlands; 5 Department of Health Services Research, Maastricht University, 6200 MD Maastricht, The Netherlands; 6 School of Health Professions Education, Maastricht University, 6200 MD Maastricht, The Netherlands; 7 Department of Neurosurgery, Canisius Wilhelmina Hospital Nijmegen, 6532 SZ Nijmegen, The Netherlands; R.droeghaag@gmail.com; 8 Department of Neurosurgery, Groningen University Medical Centre, 9700 RB Groningen, The Netherlands; 9 Department of Orthopaedic Surgery, VieCuri Medical Centre, 5900 BX Venlo, The Netherlands

**Keywords:** qualitative analysis, lumbar interbody fusion, process evaluation, patient satisfaction, qualitative research

## Abstract

*Background and Objectives*: Only limited qualitative research concerning instrumented spine surgeries has been published, despite the increasing number of these surgeries and the evident importance of qualitative analysis of the processes surrounding these complex interventions. Current qualitative research is mainly limited to the experiences, emotions and expectations of patients. Insight into the full process, including experiences from the perspective of informal caregivers and healthcare professionals, remains scarce. *Materials and Methods*: Data were gathered by means of semi-structured face-to-face interviews. In total, there were 27 participants, including 11 patients, 7 informal caregivers and 9 healthcare professionals. The interview process was audiotaped, and each interview was transcribed verbatim. To systematically analyse the gathered data, software for qualitative analysis (NVivo) was used. After immersion in the raw data of transcripts and field notes, a list of broad categories for organising the data into meaningful clusters for analysis was developed. All interviews were coded by the first author, and 25% was independently assessed by the second author. *Results:* The results of our study describe several promoting and limiting factors concerning the process of lumbar fusion surgery from the perspective of patients, informal caregivers and healthcare providers. The most frequently mentioned promoting factors were: information and opportunities to ask questions during consultations; multidisciplinary consultations; good communication and guidance during hospitalization; and follow-up appointments. The most frequently mentioned limiting factors were: lack of educational material; lack of guidance and communication prior to, during and after hospitalisation. *Conclusion:* Overall, participants were satisfied with the current healthcare-process in lumbar fusion surgery. However, we found that lack of educational material and guidance during the process led to insecurity about complaints, surgery and recovery. To improve the process of lumbar interbody fusion and to increase patient satisfaction, healthcare providers should focus on guiding and educating patients and informal caregivers about the pre-operative trajectory, the surgery and the recovery. From the healthcare providers’ perspective, the process could be improved by multidisciplinary consultations and a dedicated spine team in the operation room. Although this study focusses on lumbar fusion surgery, results could be translated to other fields of spine surgery and surgery in general.

## 1. Introduction

Since 1980, the global population of people older than 60 years has doubled. This number is expected to double again by 2050 [1]. Ageing of the population is one of the most prominent factors by which the number of instrumented spine surgeries has increased and will increase even further in the future [2,3]. Therefore, it is of significant importance to analyse, evaluate and eventually optimise the efficiency of the healthcare process and patient satisfaction [4].

Despite the increasing number of instrumented spine surgeries and the evident importance of qualitative analysis of the processes surrounding complex interventions, only limited qualitative research has been published concerning this subject. Current qualitative research is mainly limited to the experiences, emotions and expectations of patients. Insight into the full process, including experiences from the perspective of informal caregivers (ICG) and healthcare professionals, remains scarce [5,6,7,8].

The aim of this study was to gain insight into the full process surrounding lumbar fusion surgery in five Dutch hospitals. Steps in the process were evaluated by patients, informal caregivers and healthcare professionals. The ultimate goal of the study was to provide insightful qualitative data that can be used to optimise the healthcare process of lumbar interbody fusion surgeries.

## 2. Materials and Methods

The qualitative analysis described in this article is part of a large randomised controlled trial on the effectiveness and cost-effectiveness of transforaminal lumbar interbody fusion (TLIF) compared to posterior lumbar interbody fusion (PLIF): the LIFT-study. The results of this study will be published elsewhere. Ethical approval was granted, and all participants provided signed informed consent before participating in the study (LIFT-study; NTR5722, Dutch Trial Register; NL54717.096.16, CCMO).

### 2.1. Recruitment and Sample Size

In total, 27 participants from five different hospitals were included, of which 11 were patients, 7 were informal caregivers and 9 were healthcare professionals (three orthopaedic surgeons, three neurosurgeons, two specialised nurses and one physician assistant).

Patients scheduled for lumbar interbody fusion surgery, as well as their informal caregivers, specialised nurses, nurse consultants, neurosurgeons and orthopaedic surgeons, were recruited to participate in this study. These groups were selected based on their unique experiences during participation in the healthcare process of fusion surgery. Patients undergoing TLIF or PLIF were recruited for the LIFT-study in five participating hospitals in the Netherlands by two researchers (RD and IC). Eligible patients were over 18 years of age and had good comprehension of verbal and written Dutch. Informal caregivers of participating patients were likewise recruited. After informed consent, patients and informal caregivers were randomly selected for an interview. The principal investigators of the LIFT-study from each participating hospital were interviewed (Zuyderland Medical Center Heerlen, Maastricht University Medical Center +, Canisius Wilhelmina Ziekenhuis, VieCuri Medical Center, University Medical Center Groningen). Furthermore, when fusion surgeries were performed by a neurosurgeon and orthopaedic surgeon, both specialists were interviewed. This was the case in two hospitals. If applicable, specialised nurses, nurse consultants or physician assistants specialised in spine surgeries likewise participated in this study. All healthcare providers gave written informed consent to participate in this study.

Inclusion started in 2017 and was completed in 2020. Inclusion stopped when both researchers agreed on the fact that the new interviews did not lead to new information or insight, only adding new instances of already existing themes without adding new ones (i.e., the point of saturation), and the included cohort had reached maximum variation with regard to the hospital where surgery was performed [9].

### 2.2. Data Collection

Data were gathered by means of semi-structured face-to-face interviews. The interviews with the patients and informal caregivers took place three months after surgery, as it was expected that patients had recovered to the extent that they could resume daily activities, including self-care. Furthermore, it was expected that the patients could reflect on the entire process without forgetting essential information. All participants were interviewed by the same researcher (IC). They could choose whether they felt more comfortable to be interviewed either in their homes or in the privacy of a consulting room within the hospital premises. During the COVID-19 pandemic, participants were interviewed through videoconferencing. The interviewer (IC) was not involved in the treatment of the patients. A semi-structured interview guide, developed prior to the start of the study, was used (Supplementary File S1). These questions were based on participant observation, with researchers attending and observing the process during outpatients’ clinics and hospital admission. Researchers obtained additional information from healthcare professionals working in this area. Open-ended questions were included to establish a general direction for the interview. The experiences of the participants were further elaborated upon by follow-up questions. The median duration of the interviews was 34 min (range 12–78). The interview process was audiotaped, and each interview was transcribed verbatim by RD and IC. To further increase credibility, all participants received transcripts of their interviews and were given the opportunity to provide feedback or additional information.

### 2.3. Data Analysis

To systematically analyse the gathered data, the NVivo Version 1.3 software for qualitative analysis was used (QSR International Pty Ltd., Doncaster, Australia ) [10]. After immersion in the raw data of transcripts and field notes, a list of broad categories (codes in NVivo), for organising the data into meaningful clusters for analysis, was developed. To evaluate the process in a comprehensible fashion, the process was categorised as “pre-hospitalisation”, “peri-hospitalisation” and “post-hospitalisation”. A detailed description of the full process is provided below. For data analysis, an iterative approached was used. At the start of data analysis, categories and subcategories were created based on the interview guide and the interviewers’ experience. During data analysis, these categories were evaluated, adjusted and complemented by two authors (RD and IC) continuously throughout the process. The final list of codes is presented in Supplementary File S2. All interviews were coded by the first author (RD). To increase objectivity, the second author (IC) went through 25% of the transcripts and independently assessed the coded data from the interviews according to the list of categories produced by the first author. In author meetings between the first and second author, the categories, subcategories and coding were discussed, and consensus was formed on how data were categorised. As all interviews were conducted in Dutch, the interview guides and reported citations were translated into English.

### 2.4. Process Description for Open Lumbar Interbody Fusion Surgeries

The process as described below is in accordance with the protocol of the randomised controlled trial on which this qualitative study is based. This description is based on participant observation in two participating hospitals. Furthermore, orthopaedic surgeons and neurosurgeons of the other three participating hospitals were asked about the regular process of lumbar fusion surgeries in their hospital. The processes of the participating hospitals were compared and combined to one uniform process description by one of the authors (IC). Before the start of the interviews, all principal investigators verified the process description.

### 2.5. Pre-Hospitalisation

Patients with lower back pain and radiculopathy or neurogenic claudication are referred to the hospital by the general practitioners (GP). Depending on the GP’s referral, the patient is examined by either a neurologist or orthopaedic surgeon. Following the first consultation, additional diagnostic tests are performed (e.g., MRI-scans, X-rays, etc.). Subsequently, another appointment with the neurologist or orthopaedic surgeon will follow to discuss the findings. If surgery is indicated, the neurologist can refer patients to the neurosurgeon or orthopaedic surgeon. The final decision to perform surgery can be made by the orthopaedic surgeon, neurosurgeon or both during a combined consultation. In some hospitals, all patients fill out various patient-reported outcome measures (PROMs) questionnaires prior to this consultation. These PROMs can be used during consultation or for research purposes. The surgeons discuss diagnosis, prognosis, conservative options and surgical options during this consultation. Furthermore, expectations, risks and outcomes are elaborated upon. If both the surgeon(s) and the patient opt for surgical intervention through lumbar interbody fusion, the patient is placed on a waiting list. Additional information about hospitalisation, surgical technique and the postoperative course can be provided by the healthcare professional. The media through which this information is provided varies per hospital and includes brochures, websites, smartphone applications and consultations with specialised nurses.

### 2.6. Hospitalisation and Surgery (Peri-Hospitalisation)

The patient is hospitalised one day prior to surgery or on the day of surgery and abstains from food and fluid with a minimum of six hours pre-operatively. The patient is then transferred to the pre-operative holding area for preparations. When the patient enters the operation room, a time-out procedure is carried out by the surgical team, where the team consists of an orthopaedic surgeon and/or neurosurgeon, an anaesthesiologist, a nurse anaesthetist, a radiology assistant, surgical nurses and in some cases surgical residents. After the time-out procedure, the patient receives antibiotic prophylaxis, is brought under general anaesthesia and is moved to prone position. Surgery is then performed.

### 2.7. Description of Surgical Technique

A detailed description of the surgical technique is available in Supplementary File S3. A midline approach is performed, exposing the posterior lumbar elements including the facet joints. Pedicle screws and rods are attached to the back of the vertebra and an interbody fusion spacer (cage) is inserted into the disc space. Autologous bone graft is placed into the interbody space and alongside the back of the vertebra.

### 2.8. Postoperative (Peri-Hospitalisation)

After surgery, the patient is transferred to the recovery room to regain consciousness from anaesthesia and receive appropriate postoperative care. A phone call is made by the surgeon to the patient’s contact person to inform about the procedure. Postoperative pain is managed through pain medication, administered either by patient-controlled analgesia (PCA) or by the nursing staff. The pain medication used varies per hospital. Once returned to the ward, patients are visited by a doctor or a physician assistant at least once every day. Patients receive deep venous thrombosis prophylaxis according to the local hospital protocol. Furthermore, standardised physical therapy is provided. During postoperative hospitalisation, position of the implants will be checked by lumbar spine X-rays. Patients are discharged from the hospital once the pain is acceptable, the wound is dry, safe mobilisation is possible and no other complications arise.

### 2.9. Post-Hospitalisation

During the first six to eight weeks following hospitalisation, patients are advised not to receive extra physical therapy. In these first weeks, functional recovery, reduction of pain and a reduced need of pain medication is expected. Patients most commonly have consultations with their surgeon at 6 weeks, three months, six months and twelve months postoperatively, although this is dependent on surgeons’ preference. These consultations are intended to monitor recovery and, if needed, provide additional care (e.g., physical therapy, rehabilitation, medication, etc.). A lumbar spine X-ray can be used to check the position of the implant during one or more of these consultations. If no additional healthcare is needed, patients can be discharged from follow-up.

## 3. Results

Twenty-seven participants were included: eleven patients (seven males, four females, age ranged from 34–74 years), seven informal caregivers, six surgeons (three orthopaedic surgeons and three neurosurgeons), two specialised nurses and one nurse consultant.

A total of 2043 fragments were coded using 34 different codes. To evaluate the process in a comprehensible fashion, results are presented in process’ subcategories: “pre-hospitalisation”, “peri-hospitalisation” and “post-hospitalisation”. Promoting and limiting factors which are discussed during interviews with patients, informal caregivers or healthcare providers are included in Table 1 and Table 2.

### 3.1. Pre-Hospitalisation

All participants reported on the importance of the pre-operative consultation. Patients, informal caregivers and healthcare professionals all stressed the importance of informing the patients about the indication of surgery, alternative surgical and non-surgical treatments, type of surgery, possible complications and post-operative expectations. Furthermore, the majority of patients reported it was important that the surgeon had sufficient time during consultations, gave clear information and gave opportunities to ask questions. Patient 2, hospital 1: *“Eventually, you get so much information, it gets hard to understand everything and keep up. But we got the chance to ask questions”*.

Many patients and informal caregivers stated that due to the amount of information, it is beneficial that family or friends can be present during the consultations. Patient 2, hospital 1: *“…some things just don’t stick, due to the amount of information”*. Additionally, all patients stressed the importance of educational material provided by the caregivers. The media through which additional information is provided varies per hospital: e.g., websites, applications for mobile phones, informational flyers. For patients, the media through which they receive the information did not matter significantly, as long as the amount and quality of the information was adequate. Patient 6, hospital 2: *“At some point questions start popping up, and you don’t remember what the doctor told you. At these moments, I could just re-read it in the educational material, and I knew what was up”*. In some patients, the lack of information led to patients searching the internet and finding incorrect information or negative patient experiences, causing insecurity about the surgery. Patient 2, hospital 3: *“You have got to be careful while searching the internet. If you keep on searching, you only find possible complications, risks… And the stories are all the same; if something goes wrong, your life is virtually over”*. Surgeons and nurses likewise reported that the information provided during consultation should be clear, and additional standardised educational material should be provided in the pre-operative trajectory. The most important aspect of informing the patient pre-operative was to manage and clarify the expectations of the surgery for both patients and informal caregivers and prevent insecurity about the surgery and recovery.

The majority of the interviewed patients had an extensive history of complaints and failed conservative treatments. Many patients stated that not only the physical complaints, but more importantly, the consequences of the complaints on their personal and social life, had a significant impact. Restrictions in social life, sometimes even leading to social isolation, were frequently mentioned due to progressive pain during walking and mobilisation in general. Almost all patients reported that long waiting times were the biggest drawback during the process.

Many patients complained about the long waiting time between consultations, and the months-long waiting lists for surgery. Patient 1, hospital 2: *(“What do you think about the waiting time?”)* “It’s horrible. You have to wait two months for a scan, two more months for a consultation with the surgeon, and after that, you still have to wait several months before you finally get the surgery!” Several healthcare providers recognised this problem. Healthcare provider 1, hospital 1: *“All these patients are in pain, and waiting three or four months is very long when you are experiencing so much pain. At some point they don’t know how to deal with it anymore. That’s very difficult for me too at times”.* Numerous patients suggested that it would be favourable to plan multiple consultations or diagnostic procedures on the same day, mainly because driving to and waiting in the hospital can cause more pain. The interviews show that this is a high burden for these patients.

Surgeons reported that it would be advantageous if they had more time for patients during their initial visit. In two hospitals, the neurosurgeon and orthopaedic surgeon perform lumbar fusion surgeries in a multidisciplinary team and likewise have a combined pre-operative consultation for all patients who are potentially indicated for surgical intervention. All these surgeons addressed the added value of this combined consultation in terms of efficiency and expertise. Furthermore, four out of six surgeons advocate multiple consultations or diagnostic procedures on the same day, which would shorten the waiting list and be more efficient for both patients and healthcare providers. The other two surgeons did not make any specific statements on this subject. Surgeon 2, hospital 2: *“Ideally, we would work together when doing consultations, so that we can consult the new patients as well as the patients visiting for follow-up and assess indications for surgery together. Thus, we can maximise our capacity and prevent multiple, unnecessary visits to the hospital”.*

Surgeons stated that the use of pre- and post-operative patient-reported questionnaires could be useful to evaluate the quality of provided treatments and for scientific research. There was no consensus whether pre-operative questionnaires could be of added value to the pre-operative consultations. One surgeon stated that looking into the questionnaires before consultation could aid in getting a general impression of the patients’ health, while another surgeon stated that all relevant information is obtained during consultation, suggesting that the clinical relevance of questionnaires is limited.

### 3.2. Peri-Hospitalisation

Among patients, there was no evident preference for hospitalisation one day prior to surgery or on the day of surgery. Surgeons preferred hospitalisation on the day of surgery. One of the most important factors during hospitalisation was communication between patients, informal caregivers and healthcare providers. According to patients and informal caregivers, information, communication and guidance are especially important in the following situations: initial admission to the ward, preparation for surgery in the holding area, introduction in the operation room and informing patients and their families after surgery. Patient 2, hospital 2: *“I would have appreciated some information in the holding and the operation room. Some more guidance in general”.* Informal caregiver 4, hospital 2: *(“Did you receive information about arrangements for informal caregivers during the surgery?”) “No, I did not receive any information. (“Did you know where you could wait, eat, drink?”) No, we just waited in the public restaurant”*. Furthermore, most patients, informal caregivers and surgeons indicated that it is desirable that the surgeon, who performed the surgery, visits the patient after the procedure.

From the surgeons’ perspective, the greatest improvements during surgery would be a dedicated spine team, including scrub nurses, radiology assistants and anaesthesiologists. Most delay during surgery is a result of insufficient experience among the team-members. In hospitals where the neurosurgeon and orthopaedic surgeon perform lumbar fusion surgeries in a multidisciplinary team, the following possible advantages were cited: shorter surgery time, less door movements, direct quality control, sharing expertise and mutual learning.

Subsequent to surgery, pain medication is given. Although pain management is an essential part of postoperative care, there appears to be a lack of standardisation among hospitals. Both patients and healthcare providers emphasised that opioids could be used, although possible side effects (e.g., constipation, somnolence, nausea), the risk of opioid addiction and the tapering off should be taken into account.

Most patients are mobilised within the first day after surgery with the help of a physical therapist. Most patients indicated that physical therapists should provide information about mobilisation and exercise during hospitalisation, preferably both in verbal and written forms. Furthermore, it is important for patients to know which exercises they must continue after discharge. Besides these exercises, treatment by a physiotherapist is not recommended by healthcare professionals in the first weeks after surgery. Patient 2, hospital 5:

“*While at the hospital, I was not informed on which type of exercises I should do at home. So, when I was discharged, I tried some exercises with my own physiotherapist. Unfortunately, that made matters worse. I did not receive educational material on the subject either, so I searched the internet and found spine-surgery-specific exercises from other hospitals*”. Surgeon 1, hospital 4: “*I would like to guide the patients during the whole process. Right now, it seems rather black and white. If you advise physical therapy, the therapists tend to give back-specific exercises, which is undesirable in the first weeks after surgery. But it would be beneficial if the patients received some additional help with mobilisation and general physical exercise in these weeks*”.

All patients received an X-ray of the lumbar spine post-operatively. Surgeons used them mainly as a baseline image for comparison, if future imaging is needed.

Patients and informal caregivers indicated that it was preferable to know what kind of difficulties they could expect in daily life when the patient was discharged. Furthermore, all patients emphasised the value of knowing who to turn to with questions. They stated that it could be beneficial if there is a dedicated contact person in the hospital for questions about the surgery and recovery. Informal caregiver 5, hospital 2: (*“If something would have gone wrong, did you know who the contact person was?”) “No, I did not know who the contact person was”.* Patient 2, hospital 2: *(“Do you mean that some more guidance would be better? Having a dedicated contact person to turn to with questions?”) “Yes, that sums it up, more guidance throughout the whole process”.* Additionally, the transfer of medical information to the patients’ general practitioner should ideally take place within the first days after discharge so that he/she is fully aware of the physical condition of the patient in case any support is necessary.

### 3.3. Post-Hospitalisation

Patients and informal caregivers reported that the first days at home were the toughest; the wound could be painful, mobilisation was challenging and most patients needed assistance with housekeeping and self-care activities involving bending, leaning or lifting heavy objects. Though informal caregivers acknowledged that they had a large share in patient-related care and additional household tasks, most did not experience the first week at home as troublesome. Many patients cited that finding the right balance between activity and rest was challenging.

Comparable to the peri-hospitalisation period, patients are in need of information, communication and guidance. Recurring topics of insecurity were pain medication, tapering off opioids, wound healing, sutures, exercise, expectation management and hospital appointments. Once again, patients preferred adequate information in verbal and written forms about these topics. A direct contact person was stated as helpful and pleasant if patients had any unanswered questions. Furthermore, it was advantageous if the general practitioner is actively involved in the post-operative care, especially for tapering off opioids. Patient 2, hospital 1: *“It is startling how fast you can get addicted to those pills, even if you are familiar with the problem”*.

Several patients, surgeons and nurses pointed out that healthcare-initiated contacts by phone, to obtain information about the patient and to answer questions, could be helpful in the first few weeks after surgery. In hospitals in which patients were actively contacted by a dedicated contact person during the first weeks after surgery, most patients made positive statements about this extra care. In hospitals in which no dedicated contact person was present, patients were more uncertain about who to turn to with questions and their recovery in general.

From the surgeons’ perspective, the follow-up appointments six weeks and three months after surgery are the most significant. If the recovery is progressing as expected, further follow-up is only needed on indication. From the patient’s perspective, reassurance and final closure of the healthcare process are the most frequently mentioned factors determining a satisfactory ending of the follow-up. Some patients mentioned that radiography could be helpful to assure that the implants were still in place, and the recovery was advancing as to be expected. Most patients agree to not having 1- and 2-year follow-up after surgery, as long as they are assured that their recovery is acceptable, and as long as they know who to contact to when problems arise.

Aside from the negative experiences and suggestions for improvement, patients were in general satisfied about the process of the lumbar fusion surgeries, including the pre-hospitalisation, peri-hospitalisation and post-hospitalisation phases. Most stated that the pre-operative pain was significantly reduced and the surgery had a positive impact on their life. Patient 2, hospital 1: *“When I returned to the hospital after the surgery, I told the surgeon he performed a miracle. Nothing more, nothing less. They make the difference between living your life and sitting on the side-line”.*

## 4. Discussion

This study is one of the first qualitative studies about the process surrounding lumbar fusion surgery, incorporating input from patients, informal caregivers and healthcare professionals from multiple hospitals. Results of our study described several promoting and limiting factors of the lumbar fusion surgery process. The most frequently mentioned promoting factors were: information and opportunities to ask questions during consultations, multidisciplinary consultations, good communication and guidance during hospitalisation and the use of several follow-up appointments. The most frequently mentioned limiting factors were: lack of educational material as well as lack of guidance and communication prior to, during and after hospitalisation.

Previous qualitative studies on lumbar fusion processes are limited. However, studies concerning some of the abovementioned promoting and limiting factors for different disciplines are available. The study of Murtagh et al., a qualitative study focussed on improving the consultation process, for instance, found that patients were more likely to ask questions if doctors actively involve patients. For example, showing and discussing scans or X-ray results during consultation [11]. Furthermore, two studies showed that cohesive teamwork resulted in improved communication between healthcare workers, decreased the number of adverse events, improved patient-related outcomes and increased work-satisfaction among the medical staff [12,13].

Although patients, informal caregivers and healthcare providers all endorse the importance of follow-up appointments after discharge, there is a lack of evidence for the need of a post-operative follow-up after spine surgery [14]. Hospital follow-up appointments do not appear to improve readmission rates or survival in general medicine patients [15]. At last, a recent review on perioperative patient satisfaction concluded that, in order to enhance patient satisfaction, healthcare providers should focus on communication and providing information [16], which is in accordance with the results of our study.

The outcomes of our study are in line with the results of a previously published study by Damsgaard et al. [6]. They focused on how patients experience their situation from the point of making the decision to undergo spinal fusion surgery, to living everyday life after spinal fusion surgery. They concluded that spinal fusion surgery initiates hope for less pain, but also creates a feeling of insecurity for life after surgery, as patients were accustomed to a life with complaints. In our study, patients likewise addressed that insecurity about the surgery and the recovery played a significant role in their experiences. In concordance with our results, Damsgaard et al. found that providing information and clear communication between patient and healthcare provider were key factors in the process from indication for surgery to recovery.

Two other qualitative studies pointed out that a long pre-operative illness process, tumultuous recovery and unfulfilled or unrealistic expectations about the surgery were frequently reported by patients undergoing spinal fusion surgery [7,8]. Our present study underlines comparable problems: insecurity about complaints, surgery and recovery. Additionally, our study provides possible solutions or suggestions for improvement based on input from patients, informal caregivers and healthcare providers; clear preoperative information about the surgery, expectations and postoperative period, both verbally and in writing (paper, websites, mobile phone applications), could be helpful. Furthermore, a dedicated healthcare professional, for example, a nurse practitioner trained in fusion surgeries, could be beneficial in the guidance of these patients. Judging from the results of this study, these solutions might increase patient satisfaction.

One suggestion for pre-operative improvements of patients and informal caregivers was planning several appointments on one day. Practically, this could be attained in the form of a “one stop shop solution”, in which several medical specialists (e.g., neurologist, neurosurgeon, orthopaedic surgeon) are consulted in succession. Another practical solution could be using dedicated diagnostic slots (e.g., MRI, CT, X-ray) on days that carrousel consultations are planned. It should be noted that in some cases, spreading different appointments over several weeks or months is unavoidable or even necessary from a healthcare perspective.

The greatest area for improvement during surgery from the surgeons’ point of view would be a dedicated spine-surgery team. In practise, this would mean that a pool of dedicated surgical nurses, radiology assistants, anaesthesiologists and nurse anaesthetists is formed. Having a dedicated spine-surgery team could lead to standardisation of the surgical process, shorter surgery time and possibly lower complication rates [17]. Furthermore, having a protocolised pain treatment during and after surgery could lead to improved pain management. Moreover, having a standardised pain treatment protocol could result in informing and educating patients more clearly on the risks and side effects of pain medication and how to taper off these medications after surgery.

Although this study focusses on lumbar fusion surgery, results can be translated to other fields of spine surgery and surgery in general. Themes such as providing information and guidance, communication and expectation management are topics known to be important in most fields of surgery. We hypothesise that the importance of the promoting and limiting factors found in this study becomes greater as the complexity of surgical interventions increase. Furthermore, multidisciplinary consultations, specialised operation room staff and a dedicated contact person (e.g., a specialised nurse) might only be feasible in highly complex care.

Part of our study was carried out during the COVID-19 pandemic. This led to some patients having follow-up appointments through either telephone or videoconference (telehealth). Surprisingly, these patients made positive comments about the use of telehealth. Some patients who were interviewed prior to the COVID-19 pandemic likewise stated that, in specific situations, telehealth is preferred. It was pointed out that it could be beneficial to talk to the doctor without the need to physically go to the hospital. This was especially the case for patients with a quick recovery. Surgeons were equally enthusiastic about the use of telehealth. Technical difficulties with the communication system did not pose a significant problem. These results were similar to the results of a large study of Isautier et al. about patient satisfaction with telehealth during the COVID-19 pandemic [18].

A possible limitation of this study is that qualitative research incorporating interviews is always susceptible to interpretation by the researchers. To limit personal interpretation and to increase reflexivity, we: (1) included participants from different perspectives and different hospitals; (2) used open-ended questions and asked for additional information; (3) anonymised and transcribed all interviews; (4) coded and analysed text files using computer software; (5) periodically reflected on the data and used codes and categories. To limit inter-observer variability, transcribing, coding and analysing were carried out by two researchers (RD and IC) [19].

Another limitation of this study is the fact that this qualitative research was carried out as a part of a randomised controlled trial conducted in multiple hospitals in the Netherlands. This led to two study-specific questions and topics being discussed in the interviews with surgeons. These fragments are not relevant nor useful for the process evaluation of lumbar interbody fusion surgery and were therefore not included in this study. Additionally, the evaluation of this healthcare process in the Netherlands might impact the possibility to generalise some findings of our study, as various strengths and limitations in the process could be specific only to the Dutch healthcare system. Vice versa, some relevant strengths and limitations which are present in healthcare systems outside of the Netherlands cannot be identified by our study due to the study design.

Furthermore, the questions used in the interviews were not validated. It is possible that the open-ended questions biased the responses of the participants in some way. However, these open-ended questions were formed based on participant observations by researchers attending and observing the process during outpatients’ clinics and hospital admission.

Strengths of this process evaluation are the relatively large number of interviews with patients, informal caregivers and healthcare providers from five hospitals [6,7,8]. By obtaining valuable insights in the perceived health, impact of illness and treatment and the experiences of patients, informal caregivers and professional healthcare providers, this study culminates in a more complete and in-depth understanding of the total process.

## 5. Conclusions

This study is one of the first qualitative studies about the process surrounding lumbar fusion surgery, incorporating input from patients, informal caregivers and healthcare professionals. Overall, all participants were satisfied with the current healthcare process in lumbar fusion surgery. However, we found that lack of educational material and guidance during the process led to insecurity about complaints, surgery and recovery. To improve the process of lumbar interbody fusion and to increase patient satisfaction, healthcare providers should focus on guiding and educating patients and informal caregivers about the pre-operative trajectory, the surgery and the recovery. This can be accomplished by informing patients and informal caregivers both verbally and in writing (paper, websites, mobile phone applications) or by a dedicated contact person (e.g., nurse practitioner). From the healthcare providers’ perspective, the process could be improved by multidisciplinary consultations and a dedicated spine team on the operation room.

## Figures and Tables

**Table 1:** Discussed promoting factors during interviews with patients, ICGs or healthcare providers.

*Promoting Factor*	*Patients*	*Informal Caregivers*	*Healthcare Professionals*
Information and opportunities to ask question during consultations	X	X	X
Multidisciplinary consultations	X	X	X
Being accompanied by an informal caregiver to consolations	X	X	
Management of expectations	X		X
Good communication and guidance during hospitalisation	X	X	X
Mobilization with support of a physical therapist subsequent to surgery	X		X
Post-operative X-ray for later comparison	X		X
Check-up appointments	X	X	X
No unnecessary check-ups after one or two years	X		X
General satisfaction during the process	X	X	X

X = Statement(s) made in compliance with mentioned factor.

**Table 2:** Discussed limiting factors during interviews with patients, ICGs or healthcare providers.

*Limiting Factor*	*Patients*	*Informal Caregivers*	*Healthcare Professionals*
Lack of educational material	X	X	X
Long history of complaints and failed conservative treatments	X		X
Long waiting times	X		X
Lack of use of pre-operative PROMs			X
Lack of guidance and communication prior to, during and after hospitalisation	X	X	X
Lack of dedicated spine-surgery team on the operation room			X
Lack of standardised pain protocols			X
Limited or delayed involvement of the general practitioner after hospital discharge	X	X	
Lack of information on tapering off opioids	X	X	

X = Statement(s) made in compliance with mentioned factor.

## Supplementary Materials

The following are available online at https://www.mdpi.com/article/10.3390/medicina58010099/s1, Supplementary File S1—Interview Guides; Supplementary File S2—List of codes for data analysis; Supplementary File S3—Detailed description of surgical technique; Table 1 and Table 2–Limiting and promoting factors.

## Abbreviations

CT	Computed Tomography
GP	General Practitioner
ICG	Informal Caregiver
MRI	Magnetic Resonance Imaging
PCA	Patient-Controlled Analgesia
PLIF	Posterior Lumbar Interbody Fusion
PROM	Patient-Reported Outcome Measure
TLIF	Transforaminal Lumbar Interbody Fusion
