# Supplementary material for: Process Evaluation of Lumbar Interbody Fusion Surgeries in Five Dutch Hospitals: A Qualitative Analysis
Source: Medicina (Kaunas). 2022 Jan 9;58(1):99. doi: 10.3390/medicina58010099 (PMC8779538; doi:10.3390/medicina58010099)
Supplement: Supplementary file 1 [file medicina-58-00099-s001.zip › medicina-1427667-supplementary/Collaborating Authors.pdf]

LIFT-Study Group Collaborators

1. Zuyderland Medical Centre, Heerlen

H. Van Santbrink, MD, PhD[1,2,4]

2. Maastricht UMC+

M.P. Ter Laak, MD, PhD[4]

3. Radboud UMC & Canisius, Nijmegen

R.H.M.A. Bartels, MD, PhD[7]

4. UMC Groningen

J.M.A. Kuijlen, MD, PhD[8]

5. VieCuri Medical Centre, Venlo

M.H.H.M. Hulsbosch, MD[9]
