# Supplementary material for: Process Evaluation of Lumbar Interbody Fusion Surgeries in Five Dutch Hospitals: A Qualitative Analysis
Source: Medicina (Kaunas). 2022 Jan 9;58(1):99. doi: 10.3390/medicina58010099 (PMC8779538; doi:10.3390/medicina58010099)
Supplement: Supplementary file 1 [file medicina-58-00099-s001.zip › medicina-1427667-supplementary/Supplemental File S1 - Interview Guides.pdf]

Additional File S1 – Interview Guides

Checklist Physician Assistant/Specialized Nurse:

- **Preoperative:**
  - Which type of patients are seen in the outpatient clinic?
    - Spondylodesis?
    - How many visits to outpatient clinic?
  - Who refers the patients to you?
  - How do you evaluate the transfer of medical information?
  - What information do you provide the patients?
    - Preoperative
      - What is the added value in comparison with consultations by a surgeon?
    - Process of hospitalization
    - Postoperative
  - Are informal caregivers and/or family involved?
  - Use of patient reported questionnaires in outpatient clinic?
  - Use of educational material?
    - Flyers
    - Websites
  - Do you see all patients who receive spodylodesis?
  - Do you get feedback form patients on this consultation?
  - What are common preoperative problems for patients?
    - How are these problems solved?
  - Do you also provide this extra care for other types of surgery?
  - Are there other things you arrange for?
    - Logistic problem solving?
  - Do you have any suggestion for improvement in the preoperative trajectory?

- **Surgery:**
  - Are patients hospitalized one day prior to surgery?
    - Patients' experiences?
    - Do you have any specific tasks during surgery?
  - Tasks on day of surgery?
  - Do you speak with informal caregivers during first hospitalization and surgery?

- **Hospitalization:**
  - What are common peri-hospitalization problems for patients?
    - Are there ways to solve these problems?
  - Tasks during hospitalization?
    - Do you assist/function as the ward physician?
    - Arragnements of post-hospitalization care?
      - Do you visit hospitalized patiens after surgery?
      - Do you arrange homecare if necessary?
  - Do you have any suggestion for improvement in the peri-hospitalization trajectory?

- **Post-hospitalization:**
  - Do you see patients in the outpatient clinic after surgery?
    - Wound assessment?
    - Other assessments?
    - How many times do you see patients in the outpatient clinic after surgery?
    - In which cases do you consult the surgeon or supervisor?
    - Is patients have question in between visits, who can they contact?
  - Frequently asked questions by patients?
    - How can we overcome/answer these questions?
  - What information do you provide the patients post-hospitalization?
    - Fragmin
    - Physical therapy
    - Exercise
    - Sutures
    - Pain medication
    - Homecare
  - Use of patient reported questionnaires in outpatient clinic?
  - Do you have any suggestion for improvement in the post-hospitalization trajectory?
  - Do informal caregivers play a significant role in the postoperative care? Do we underestimate the amount of help informal caregivers provide?
  - Do patients report on common problems?
    - Subsequent to surgery
    - Long term
    - Possible solutions?
  - Do you have any suggestion for improvement in the post-hospitalization trajectory?

Checklist patients:

- **Preoperative:**
- Visits to outpatient clinic before surgery:
  - How many visits?
    - Transfer of medical information
    - Waiting time
  - What information did you receive?
    - Different treatment options
    - Complications
    - Expected period of recovery
    - Expectations regarding treatment (leg pain vs. backpain)
    - Was the surgical intervention clearly explained?
      - What will be done?
      - How long will the surgery take?
      - General anesthesia
      - Pain medication with morphine pump or oral medication
  - Use of educational material
    - Flyer
    - Websites

    - Were all consultations useful?
    - Did you remember the important information?
        - Were difficult terms used, was everything explained in an understandable fashion?
    - Were there enough opportunities to ask questions?
    - Do you have any suggestion for improvement in the pre-hospitalization trajectory?
- **Surgery:**
    - General remarks about day of surgery?
        - How did you experience the admission to the ward?
        - How were you prepared for surgery?
        - How did you experience the transfer to the holding?
        - How did you experience the preparations for anesthesia?
        - How did you experience the interaction in the operating room prior to surgery?
            - Accompaniment
            - Medical information transfer
            - Identification of patient
            - Was it clear why everyone was there and what everyone's role was during surgery?
    - Positive/negative experiences?
    - Do you have any suggestion for improvement?
- **Hospitalization:**
    - How long did you have to stay hospitalized?
    - Experiences?
        - Which doctors did you see during hospitalization?
            - Ward doctor, surgeon?
            - Responsible surgeon(s)?
        - Experience with different doctors
        - Was it clear to you what role each healthcare professional had?
    - Go through protocol:
        - Wound examination
        - Physical therapy (Day after surgery, climbing stairs, educational material about physical therapy)
        - Pain medication (pump, sufficient, pain management, tapering off medication)
        - Fragmin (6 weeks?)
        - X-ray of the lumbar spine during hospitalization?
        - Corset?
    - Information about post-hospitalization trajectory and homecare arranged if necessary?
        - Fragmin
        - Physical therapy
        - Exercise
        - Sutures
        - Pain medication

        - Homecare
        - Medical rehabilitation
    - Did you remember the important information?
        - Were difficult terms used, was everything explained in an understandable fashion?
    - Were there enough opportunities to ask questions?
    - Do you have any suggestion for improvement during hospitalization?
- **<u>Post-hospitalization:</u>**
    - What were the first days at home?
    - Did you need any help?
        - Medication (Pain medication, fragmin).
        - Physical therapy
        - Homecare
        - Medical rehabilitation
        - Sufficient care?
    - Setting goals and achieving them (what were the biggest obstacles or barriers)
        - Did you recover as you expected?
    - How many times do you visit the outpatient clinic after surgery?
        - Wound examinations?
        - Visits with the responsible surgeon
            - Recovery
            - Goals and difficulties
            - X-ray
            - Follow-up appointments
    - Useful?
    - Did you remember the important information?
        - Were difficult terms used, was everything explained in an understandable fashion?
    - Do you have any suggestion for improvement during hospitalization?
    - Do you think outpatient visits 6 and 12 months after surgery are useful?

Checklist informal caregivers:

- **<u>Preoperative:</u>**
    - Visits to outpatient clinic before surgery
    - Were you present at all appointments?
    - What are your experiences?
    - What information did you receive?
        - Different treatment options
        - Complications
        - Expected period of recovery
        - Expectations regarding treatment (leg pain vs. backpain)
        - Was the surgical intervention clearly explained?
            - What will be done?

            - How long will the surgery take?
            - General anesthesia
            - Pain medication with morphine pump or oral medication
    - Were all consultations useful?
    - Did you remember the important information?
        - Were difficult terms used, was everything explained in an understandable fashion?
    - Were there enough opportunities to ask questions?
    - Do you have any suggestion for improvement in the pre-hospitalization trajectory?
- **Surgery:**
    - Were you informed about the surgery?
        - Communication
    - How did you experience the day of surgery?
        - Which services were provided?
        - Were you informed about services you could use?
    - Do you have any suggestion for improvement?
- **Hospitalization:**
    - Experiences?
        - Which doctors did you see during hospitalization?
            - Ward doctor, surgeon?
            - Responsible surgeon(s)?
        - Experience with different doctors
        - Was it clear to you what role each healthcare professional had?
    - Did you receive information about the post-operative care?
        - Were you involved in the postoperative care?
    - Useful services for informal caregivers?
        - Were you informed about services you could use?
        - What went well?
        - What would have been useful?
    - Do you have any suggestion for improvement?
- **Post- hospitalization:**
    - Did you have a significant role during recovery?
    - Did you receive enough support/help with providing care (e.g. fragmin)?
    - Do we underestimate the amount of help informal caregivers provide?
    - Were you present at postoperative outpatient clinic visits?
    - Experiences?
    - Useful?
    - Did you remember the important information?
        - Were difficult terms used, was everything explained in an understandable fashion?
    - Were there enough opportunities to ask questions?
    - Do you have any suggestion for improvement in the post-hospitalization trajectory?
    - Do you think outpatient visits 6 and 12 months after surgery are useful?

Checklist surgeons:

- **Preoperative:**
    - How many times do you see a patient before surgery?
    - How much time do you have every visit?
    - What information did you give the patients?
        - Different treatment options
        - Complications
        - Expected period of recovery
        - Expectations regarding treatment (leg pain vs. backpain)
    - Patient reported questionnaires in outpatient clinic?
        - Do you use them?
        - Are they useful?
        - Do you have questionnaires on the pain experience?
    - Use of educational material?
        - Flyers
        - Websites
    - Are all visits useful?
    - Do you have any suggestion for improvement in the pre-hospitalization trajectory?
- **Surgery:**
    - Preference for TLIF or PLIF?*
        - Why?
    - Would you consider choosing the most cost-effective alternative?*
        - Why?
    - How do you plan surgeries? How do you optimize the process and use time effectively?
        - Do you perform the surgery alone or with two surgeons (neurosurgeon/orthopedic surgeon)?
            - Room for improvement?
        - Time schedule of surgery, including anesthetist and radiology
            - How long does the surgery take?
            - How do you manage these types of surgery?
            - Room for improvement?
        - Radiology
            - Which system is used?
            - When is radiology performed? How long does it take?
            - How do you manage the imaging?
            - Room for improvement?
        - Door movement?
            - Strict policy
            - Room for improvement?
- **(Post) Hospitalization:**
    - How many times and at what interval do you want patients to visit the outpatient clinic after surgery?
        - Wound examinations?

        - How is this interval chosen?
        - Is this recorded in protocols?
        - Do you use (radiographic) imaging? When and why?
    - Visits 1- and 2-year post-operative?
        - Why or why not??
        - Useful?
    - Questionnaires
        - Useful?
        - How do you use the outcomes?
    - How is the post-hospitalization care arranged?
        - Do you visit the hospitalized patient after surgery?
        - Which types of additional care are needed (e.g. physical therapy)?
    - Do you have any suggestion for improvement in the (post)hospitalization trajectory?

* Study-specific questions. Fragments regarding these questions are not relevant nor useful for the process evaluation of lumbar interbody fusion surgery, and were therefor not included in this study.
