# Supplementary material for: Process Evaluation of Lumbar Interbody Fusion Surgeries in Five Dutch Hospitals: A Qualitative Analysis
Source: Medicina (Kaunas). 2022 Jan 9;58(1):99. doi: 10.3390/medicina58010099 (PMC8779538; doi:10.3390/medicina58010099)
Supplement: Supplementary file 1 [file medicina-58-00099-s001.zip › medicina-1427667-supplementary/Supplemental File S3 ¿C Detailed description of surgical technique.pdf]

Additional File S3 - Detailed description of surgical technique

Description of surgery

A midline approach is performed, exposing the posterior lumbar elements including the facet joints. Pedicle screws are placed bilaterally, using fluoroscopic guidance or navigation, depending on preference of the surgeon.

TLIF: Unilateral exposure to the intervertebral disc is assured by unilateral facetectomy, decompressing the descending and leaving roots. In case of bilateral symptomatic leg pain, the side of the unilateral approach is free of choice for the surgeon. Unilateral facetectomy is performed to gain access to the intervertebral disc. After removal of the intervertebral disc, endplate cartilage is prepared to provide a host bed of bleeding subchondral bone for placement of the intercorporal cage. The TLIF cage size is determined by a trial cage and checked by fluoroscopy. The definitive cage is filled with autologous bone or allograft and is tamped into place. Its final position is checked radiological. After placement of the TLIF cage, the remainder of the disc space is filled with autologous bone, obtained from the decompression. A titanium rod interconnects the screws on each side. In several hospitals, epidural analgesia is administered. The spreader is removed and the wound is thoroughly irrigated and closed in several layers without suction drainage.

PLIF: Bilateral access to the intervertebral disc assured by medial facetectomy. The intervertebral disc will be removed bilaterally. Subsequently, endplate cartilage is prepared to provide a host bed of bleeding subchondral bone for placement of the cages. Before placement of the definitive cages, the disc space is partially filled with autologous bone, obtained from decompression. Cages will be placed bilateral after determination of cage size by trail cages and fluoroscopy. These are also filled with autologous bone or allograft and are tamped into place with fluoroscopic guidance. Their position is checked radiological. A titanium rod interconnects the screws on each side. In several hospitals, epidural analgesia is administered. The spreader is removed and the wound is thoroughly irrigated and closed in several layers without suction drainage.
