# Supplementary material for: Process Evaluation of Lumbar Interbody Fusion Surgeries in Five Dutch Hospitals: A Qualitative Analysis
Source: Medicina (Kaunas). 2022 Jan 9;58(1):99. doi: 10.3390/medicina58010099 (PMC8779538; doi:10.3390/medicina58010099)
Supplement: Supplementary file 1 [file medicina-58-00099-s001.zip › medicina-1427667-supplementary/Tables S1 & S2 - Limiting and promoting factors.pdf]

**Tables S1 & S2 – Limiting and promoting factors**

*Table S1 - Discussed promoting factors during interviews with patients, ICGs or healthcare providers.*

| *Promoting factor* | *Patients* | *Informal caregivers* | *Healthcare professionals* |
|---|---|---|---|
| Information and opportunities to ask question during consultations | X | X | X |
| Multidisciplinary consultations | X | X | X |
| Being accompanied by an informal caregiver to consolations | X | X | |
| Management of expectations | X | | X |
| Good communication and guidance during hospitalisation | X | X | X |
| Mobilization with support of a physical therapist subsequent to surgery | X | | X |
| Post-operative X-ray for later comparison | X | | X |
| Check-up appointments | X | X | X |
| No unnecessary check-ups after one or two years | X | | X |
| General satisfaction during the process | X | X | X |

X = Statement(s) made in compliance with mentioned factor.

*Table S2 - Discussed limiting factors during interviews with patients, ICGs or healthcare providers.*

| *Limiting factor* | *Patients* | *Informal caregivers* | *Healthcare professionals* |
|---|---|---|---|
| Lack of educational material | X | X | X |
| Long history of complaints and failed conservative treatments | X | | X |
| Long waiting times | X | | X |
| Lack of use of pre-operative PROMs | | | X |
| Lack of guidance and communication prior to, during and after hospitalisation | X | X | X |
| Lack of dedicated spine-surgery team on the operation room | | | X |
| Lack of standardised pain protocols | | | X |
| Limited or delayed involvement of the general practitioner after hospital discharge | X | X | |
| Lack of information on tapering off opioids | X | X | |

X = Statement(s) made in compliance with mentioned factor.
