# Supplementary figures and images for: Process Evaluation of Lumbar Interbody Fusion Surgeries in Five Dutch Hospitals: A Qualitative Analysis
Source: Medicina (Kaunas). 2022 Jan 9;58(1):99. doi: 10.3390/medicina58010099 (PMC8779538; doi:10.3390/medicina58010099)

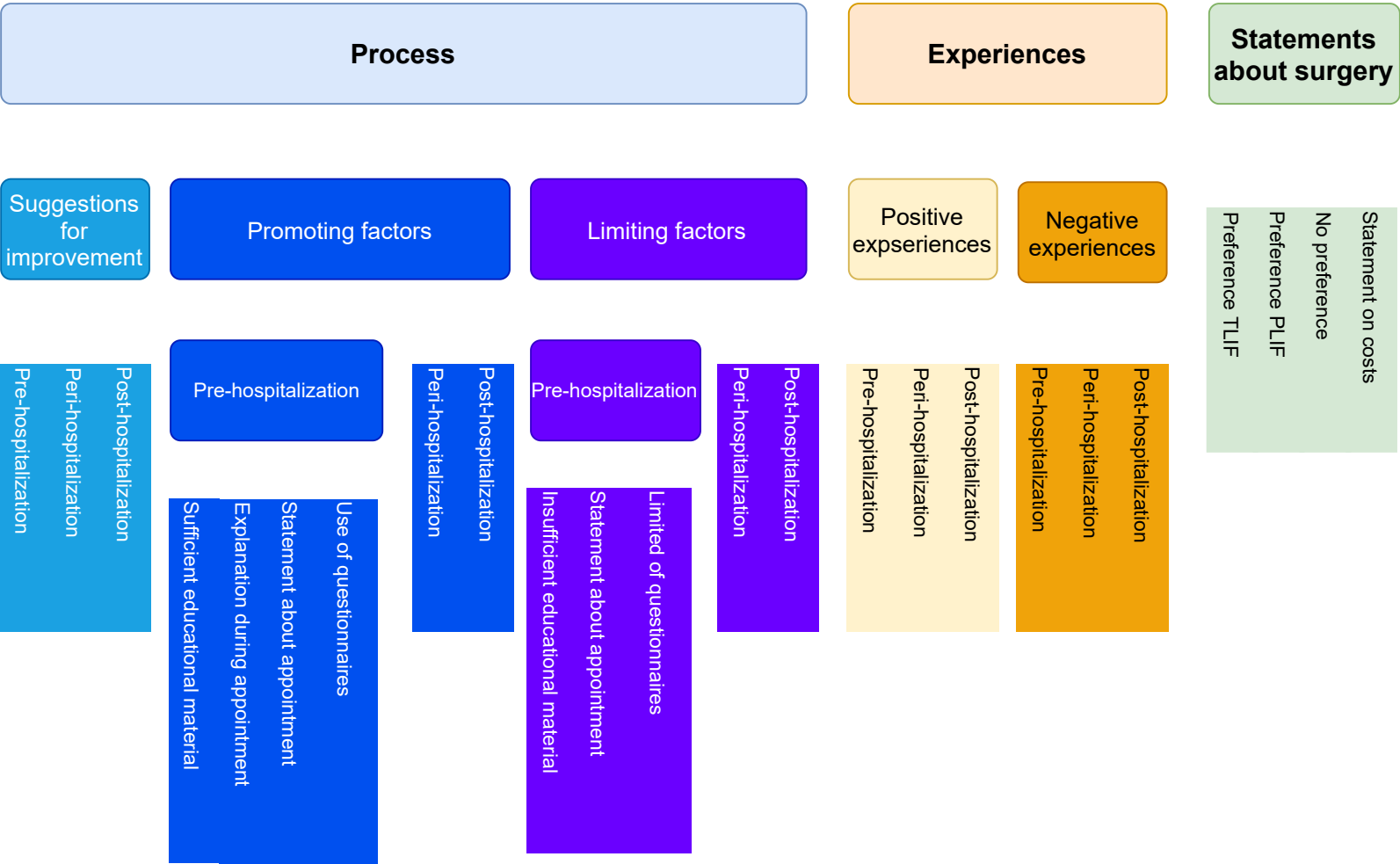

Supplement: Supplementary file 1 [file medicina-58-00099-s001.zip › medicina-1427667-supplementary/Supplemental File S2 - List of codes for data-analysis.pdf]
